# (3,5,5,6,8,8-Hexamethyl-5,6,7,8-tetra­hydro­naphthalen-2-yl)methanol: a possible metabolite of the synthetic musk fragrance AHTN

**DOI:** 10.1107/S1600536811018009

**Published:** 2011-05-20

**Authors:** Rüdiger Faust, Djawed Nauroozi, Clemens Bruhn, Britta Koch, Paul Kuhlich, Christian Piechotta, Irene Nehls

**Affiliations:** a Institute for Chemistry, University of Kassel, Heinrich-Plett-Strasse 40, 34132 Kassel, Germany; b BAM–Federal Institute for Materials Research and Testing, Richard-Willstätter-Strasse 11, 12489 Berlin, Germany

## Abstract

The title compound (AHTN-OH), C_17_H_26_O, was prepared in order to provide standard materials for the qualitative and quanti­tative analysis of environmental pollutants. The mol­ecule possesses a chiral C atom, although the structure determination was performed on racemic material, expressed in the structure as disordered chiral sites. The asymmetric unit consists of four AHTN-OH mol­ecules containing an hy­droxy group and forming a tetra­meric cyclic motif built up by four strong hydrogen bonds between these hy­droxy groups and additionally by two weak C—H⋯π inter­actions. Furthermore, these tetra­mers are linked *via* very weak C—H⋯π inter­actions, forming chains along the *c* axis.

## Related literature

For the solid state structure of AHTN, see De Ridder *et al.* (1990 ▶); for the partially oxidized derivatives AHTN-COOH, see: Kuhlich *et al.* (2010 ▶) and AHTN-CHO, see: De Ridder *et al.* (1994 ▶). The accumulation potential of AHTN in humans, wildlife and the environment is of concern (Lucken­bach & Epel, 2005 ▶; Martin *et al.*, 2007 ▶), especially as it has been shown to act as an endocrine disruptor (Luckenbach & Epel, 2005 ▶). For our efforts to provide standard materials for the qualitative and quanti­tative analysis of food contam­in­ants, see: Siegel *et al.* (2009 ▶). For structure–fragrance relationships, see: Amoore (1970 ▶). Relationships of this kind increasingly take into account structural design elements such as mol­ecular sites of hydro­philicity or inter­molecular inter­actions such as hydrogen bonds, see: Beets (1957 ▶, 1978 ▶); Dravnieks & Laffort (1972 ▶). The title compound was obtained by the oxidation of the AHTNs acyl side chain to a carb­oxy­lic acid, followed by carboxyl reduction to the primary alcohol, see: Valdersnes *et al.* (2006 ▶). 
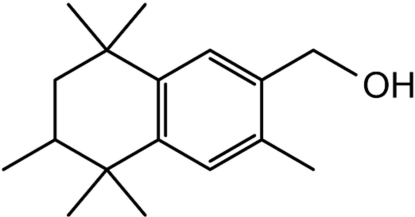


## Experimental

### 

#### Crystal data

C_17_H_26_O
*M* *_r_* = 246.38
Orthorhombic, 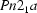

*a* = 17.6364 (7) Å
*b* = 29.0183 (15) Å
*c* = 11.6701 (5) Å
*V* = 5972.5 (5) Å^3^
*Z* = 16
Mo *K*α radiation
μ = 0.07 mm^−1^
*T* = 120 K
0.60 × 0.60 × 0.07 mm

#### Data collection

Stoe IPDS 2 diffractometer
Absorption correction: integration (*X-RED32*; Stoe & Cie, 2005 ▶) *T*_min_ = 0.955, *T*_max_ = 0.994
23401 measured reflections
5436 independent reflections
4395 reflections with *I* > 2σ(*I*)
*R*_int_ = 0.082

#### Refinement

*R*[*F* ^2^ > 2σ(*F* ^2^)] = 0.061
*wR*(*F* ^2^) = 0.173
*S* = 1.07
5436 reflections
708 parameters
25 restraints
H-atom parameters constrained
Δρ_max_ = 0.54 e Å^−3^
Δρ_min_ = −0.41 e Å^−3^

### 

Data collection: *X-AREA* (Stoe & Cie, 2005 ▶); cell refinement: *X-AREA*; data reduction: *X-RED32* (Stoe & Cie, 2005 ▶); program(s) used to solve structure: *SHELXS97* (Sheldrick, 2008 ▶); program(s) used to refine structure: *SHELXL97* (Sheldrick, 2008 ▶); molecular graphics: *ORTEP-3* (Farrugia, 1997 ▶); software used to prepare material for publication: *SHELXL97*.

## Supplementary Material

Crystal structure: contains datablocks I, global. DOI: 10.1107/S1600536811018009/bg2389sup1.cif

Structure factors: contains datablocks I. DOI: 10.1107/S1600536811018009/bg2389Isup2.hkl

Supplementary material file. DOI: 10.1107/S1600536811018009/bg2389Isup3.cml

Additional supplementary materials:  crystallographic information; 3D view; checkCIF report

## Figures and Tables

**Table 1:** Hydrogen-bond geometry (Å, °) *Cg*4, *Cg*10, *Cg*14 and *Cg*17 are the centroids of the C19–C22/C27/C28, C36–C39/C44/C45, C53–C56/C61/C62 and C2–C5/C10/C11 rings, respectively.

*D*—H⋯*A*	*D*—H	H⋯*A*	*D*⋯*A*	*D*—H⋯*A*
O1—H1⋯O4	0.84	1.93	2.753 (5)	168
O2—H2⋯O1	0.84	1.98	2.794 (4)	162
O3—H3⋯O2	0.84	1.92	2.7545)	174
O4—H4⋯O3	0.84	1.95	2.790 (4)	173
C29—H29*A*⋯*Cg*14^i^	0.98	2.83	3.632 (4)	140
C29—H29*B*⋯*Cg*17	0.98	2.58	3.466 (5)	151
C63—H63*A*⋯*Cg*10	0.98	2.69	3.471 (5)	137
C63—H63*B*⋯*Cg*4^ii^	0.98	2.72	3.641 (5)	157

Symmetry codes: (i) 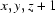; (ii) 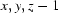.

**Fig. 1.**
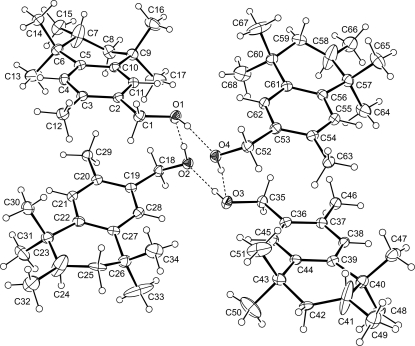
Tetrameric motif of four hydrogen bonded molecules of 1 in the crystal. The ellipsoids represent the 30% probability level. The H atoms are drawn as circles of arbitrary radius, only one split position of the atoms C8, C25, C42 and C59 is displayed.

## supplementary crystallographic information

### Comment

6-Acetyl-1,1,2,4,4,7-hexamethyltetraline (AHTN, Tonalide*^R^* is a member of a group of synthetic fragrances designed to emulate the scent of natural musk. It is widely used in perfumes, cosmetics, detergents and air fresheners and hence is produced in several thousand tons per year worldwide. However, its accumulation potential in humans, wildlife and in the environment currently gives rise to concern, (Luckenbach & Epel, 2005; Martin *et al.*, 2007) in particular in view of the fact that AHTN has been shown to act as an endocrine disruptor by inhibiting the cellular xenobiotic defense system (Luckenbach & Epel, 2005). A possible metabolite of the lipophilic AHTN is the title compound (3,5,5,6,8,8-hexamethyl-5,6,7,8-tetrahydronaphthalene-2-yl)methanol 1 (AHTN-OH), formally obtained by the oxidation of the AHTNs acyl side chain to a carboxylic acid followed by carboxyl reduction to the primary alcohol (Valdersnes *et al.*, 2006). In our continuing effort to provide standard materials for the qualitative and quantitative analysis of food contaminants (Siegel *et al.*, 2009) and environmental pollutants, we have elucidated the crystal structure of 1. These data will assist, for example, fragrance designers in establishing structure-fragrance relationships (Amoore, 1970; Beets, 1978). Relationships of this kind increasingly take into account structural design elements such as molecular sites of hydrophilicity or intermolecular interactions such as hydrogen bonds (Beets, 1957; Dravnieks & Laffort, 1972). The solid state structures of AHTN, (De Ridder *et al.*, 1990) and of the partially oxidized derivatives AHTN-COOH (Kuhlich *et al.*, 2010) and AHTN-CHO (De Ridder *et al.*, 1994) have been reported.

AHTN-OH 1 crystallizes in an orthorhombic unit cell of the non-centrosymmetric space group P n 2_1_ a. The asymmetric unit contains 4 formular units that are bonded *via* four strong hydrogen bonds, forming a four-membered cyclic arrangement (Table 1). No further intermolecular bonds, hydrogen bonds or other strong interactions between these tetrameric units can be observed. Since the starting material AHTN was used as a racemic mixture in the synthesis of 1, 1 is found to occur as a racemic mixture in the crystal. In the present case both enantiomers are represented by the split positions of the methylene groups at C8, C25, C42 and C59 (only one split position is shown in Fig. 1). Each split position determines the *R* or S configuration of the adjacent atoms C7, C24, C41 and C58, respectively. The present study does therefore not allow a correlation between the olfactoric qualities and the absolute configuration of the two enantiomers of 1. It is evident, however, that the vapor pressure (*cf.* the relatively high mp. of 1) and hence the fragrance intensity of 1 is significantly reduced compared to related compounds such as the parent AHTN, AHTN-CHO and AHTN-COOH, which are less engaged in hydrogen bonded networks in the solid state.

### Experimental

A solution of 3,5,5,6,8,8-hexamethyl-5,6,7,8-tetrahydronaphthalene-2- carboxylic acid (AHTN-COOH, 913 mg, 3.5 mmol) (Kuhlich *et al.*, 2010) in dry diethylether (100 ml) was added (1 h) at 50 °C to a stirred suspension of LiAlH_4_ (540 mg, 14.2 mmol) in diethylether (40 ml). After 0.5 h the cooled (0 °C) reaction mixture was carefully hydrolyzed with H_2_O (120 ml) and acidified with 6 *M* HCl solution (90 ml). The mixture was extracted with CH_2_Cl_2_ (90 ml), the organic phase dried over MgSO_4_, filtered and the solvent was evaporated under reduced pressure. Crystallization from ethyl acetate followed by column chromatography (SiO_2_, CHCl_3_/MeOH 3:1) gave the product as colorless solid [m.p. 111 °C]. Single crystals of (3,5,5,6,8,8-hexamethyl-5,6,7,8-tetrahydronaphthalen-2-yl)methanol suitable for X-ray diffraction were obtained by slow evaporation of an ethyl acetate solution (Valdersnes *et al.*, 2006).

### Refinement

The structure shows disorder, which could be resolved at positions C8/C8a, C25/C25*a*, C42/C42*a* and C59/C59*a*. The split positions were included to the model with fixed occupancy parameters of 0.5. Hydrogen atoms at the positions depicted with "a" were not included to the model. The expectable split positions of the adjacent atoms could not be refined.

The carbon bonded H atoms were placed in calculated positions (C-H:0.95-0.99Å) and refined according to the riding model with the 1.2 fold isotropic displacement parameter of their bonding partner. The H atoms of the OH groups were refined according to the rotating group routine (O-H: 0.85Å) and ended up in the expected positions for the O—H···O hydrogen bonds.

### Crystal data

**Table:**

C_17_H_26_O	*F*(000) = 2176
*M**_r_* = 246.38	*D*_x_ = 1.096 Mg m^−^^3^
Orthorhombic, *P**n*2_1_*a*	Mo *K*α radiation, λ = 0.71073 Å
Hall symbol: P -2ac -2n	Cell parameters from 20214 reflections
*a* = 17.6364 (7) Å	θ = 1.4–25.6°
*b* = 29.0183 (15) Å	µ = 0.07 mm^−^^1^
*c* = 11.6701 (5) Å	*T* = 120 K
*V* = 5972.5 (5) Å^3^	Plate, colourless
*Z* = 16	0.60 × 0.60 × 0.07 mm

### Data collection

**Table:**

Stoe IPDS 2 diffractometer	5436 independent reflections
Radiation source: sealed X-ray tube, 12 x 0.4 mm long-fine focus	4395 reflections with *I* > 2σ(*I*)
plane graphite	*R*_int_ = 0.082
Detector resolution: 6.67 pixels mm^-1^	θ_max_ = 25.2°, θ_min_ = 1.9°
rotation method scans	*h* = −20→21
Absorption correction: integration (*X-RED*; Stoe & Cie, 2005)	*k* = −30→34
*T*_min_ = 0.955, *T*_max_ = 0.994	*l* = −13→12
23401 measured reflections	

### Refinement

**Table:**

Refinement on *F*^2^	Primary atom site location: structure-invariant direct methods
Least-squares matrix: full	Secondary atom site location: difference Fourier map
*R*[*F*^2^ > 2σ(*F*^2^)] = 0.061	Hydrogen site location: inferred from neighbouring sites
*wR*(*F*^2^) = 0.173	H-atom parameters constrained
*S* = 1.07	*w* = 1/[σ^2^(*F*_o_^2^) + (0.109*P*)^2^ + 1.1745*P*] where *P* = (*F*_o_^2^ + 2*F*_c_^2^)/3
5436 reflections	(Δ/σ)_max_ = 0.001
708 parameters	Δρ_max_ = 0.54 e Å^−^^3^
25 restraints	Δρ_min_ = −0.41 e Å^−^^3^

### Special details

**Table:**

Geometry. All e.s.d.'s (except the e.s.d. in the dihedral angle between two l.s. planes) are estimated using the full covariance matrix. The cell e.s.d.'s are taken into account individually in the estimation of e.s.d.'s in distances, angles and torsion angles; correlations between e.s.d.'s in cell parameters are only used when they are defined by crystal symmetry. An approximate (isotropic) treatment of cell e.s.d.'s is used for estimating e.s.d.'s involving l.s. planes.

### Fractional atomic coordinates and isotropic or equivalent isotropic displacement parameters (Å^2^)

**Table:**

	*x*	*y*	*z*	*U*_iso_*/*U*_eq_	Occ. (<1)
C1	1.0423 (2)	0.11509 (17)	0.0704 (4)	0.0325 (10)	
H1B	1.0832	0.1050	0.0178	0.039*	
H1A	1.0251	0.0878	0.1141	0.039*	
C2	1.0733 (2)	0.15044 (16)	0.1521 (4)	0.0279 (9)	
C3	1.1318 (2)	0.13801 (17)	0.2277 (4)	0.0289 (9)	
C4	1.1599 (2)	0.17084 (17)	0.3015 (4)	0.0300 (9)	
H4A	1.2006	0.1624	0.3506	0.036*	
C5	1.1318 (2)	0.21608 (17)	0.3082 (4)	0.0314 (10)	
C6	1.1672 (2)	0.24844 (17)	0.3991 (4)	0.0359 (10)	
C7	1.1305 (4)	0.2969 (2)	0.3948 (7)	0.087 (3)	
H7	1.1591	0.3118	0.3307	0.105*	
C8	1.0509 (3)	0.2989 (2)	0.3519 (5)	0.0421 (17)	0.70
H8A	1.0360	0.3317	0.3461	0.051*	0.50
H8B	1.0177	0.2844	0.4099	0.051*	0.50
C8A	1.0866 (10)	0.3115 (4)	0.2919 (12)	0.060 (5)*	0.30
C9	1.0355 (2)	0.27644 (16)	0.2392 (4)	0.0363 (10)	
C10	1.0722 (2)	0.22849 (16)	0.2345 (4)	0.0287 (9)	
C11	1.0453 (2)	0.19542 (17)	0.1564 (4)	0.0315 (9)	
H11A	1.0064	0.2040	0.1044	0.038*	
C12	1.1617 (3)	0.08893 (17)	0.2306 (4)	0.0355 (10)	
H12A	1.1854	0.0815	0.1569	0.043*	
H12B	1.1197	0.0676	0.2449	0.043*	
H12C	1.1994	0.0860	0.2919	0.043*	
C13	1.1584 (4)	0.2263 (3)	0.5159 (5)	0.0618 (17)	
H13A	1.1752	0.1941	0.5121	0.074*	
H13B	1.1051	0.2274	0.5391	0.074*	
H13C	1.1893	0.2430	0.5719	0.074*	
C14	1.2516 (3)	0.25524 (19)	0.3718 (4)	0.0389 (11)	
H14A	1.2570	0.2677	0.2942	0.047*	
H14B	1.2779	0.2255	0.3767	0.047*	
H14C	1.2738	0.2768	0.4271	0.047*	
C15	1.1490 (3)	0.3283 (2)	0.4946 (5)	0.0536 (14)	
H15A	1.2034	0.3351	0.4946	0.064*	
H15B	1.1354	0.3129	0.5665	0.064*	
H15C	1.1203	0.3570	0.4873	0.064*	
C16	1.0551 (4)	0.3039 (2)	0.1311 (6)	0.0597 (17)	
H16A	1.1101	0.3080	0.1262	0.072*	
H16B	1.0305	0.3342	0.1345	0.072*	
H16C	1.0371	0.2872	0.0634	0.072*	
C17	0.9491 (3)	0.2727 (2)	0.2425 (8)	0.080 (3)	
H17A	0.9269	0.3036	0.2471	0.096*	
H17B	0.9337	0.2547	0.3097	0.096*	
H17C	0.9311	0.2573	0.1728	0.096*	
C18	0.8604 (2)	0.14025 (18)	0.2550 (4)	0.0341 (10)	
H18B	0.8111	0.1439	0.2946	0.041*	
H18A	0.8916	0.1677	0.2730	0.041*	
C19	0.9001 (2)	0.09740 (16)	0.3011 (4)	0.0284 (9)	
C20	0.9564 (2)	0.10026 (16)	0.3867 (4)	0.0306 (9)	
C21	0.9910 (2)	0.05958 (17)	0.4202 (4)	0.0323 (9)	
H21A	1.0298	0.0614	0.4765	0.039*	
C22	0.9727 (2)	0.01548 (17)	0.3769 (4)	0.0309 (9)	
C23	1.0186 (3)	−0.02688 (18)	0.4187 (4)	0.0367 (10)	
C24	0.9901 (4)	−0.0705 (2)	0.3630 (8)	0.098 (3)	
H24	0.9470	−0.0762	0.4170	0.118*	
C25	0.9444 (4)	−0.0682 (3)	0.2578 (6)	0.042 (3)	0.50
H25A	0.9209	−0.0988	0.2459	0.051*	0.50
H25B	0.9794	−0.0629	0.1928	0.051*	0.50
C25A	0.9119 (5)	−0.0724 (2)	0.3193 (11)	0.051 (3)	0.50
C26	0.8838 (3)	−0.03345 (17)	0.2509 (4)	0.0420 (12)	
C27	0.9135 (2)	0.01311 (17)	0.2964 (4)	0.0307 (9)	
C28	0.8802 (2)	0.05400 (18)	0.2594 (4)	0.0324 (10)	
H28A	0.8417	0.0522	0.2026	0.039*	
C29	0.9786 (2)	0.14571 (17)	0.4411 (4)	0.0324 (10)	
H29A	0.9335	0.1604	0.4739	0.039*	
H29B	1.0008	0.1659	0.3829	0.039*	
H29C	1.0158	0.1402	0.5019	0.039*	
C30	1.1019 (3)	−0.0198 (2)	0.3880 (5)	0.0467 (12)	
H30A	1.1199	0.0092	0.4216	0.056*	
H30B	1.1073	−0.0185	0.3045	0.056*	
H30C	1.1319	−0.0455	0.4182	0.056*	
C31	1.0124 (3)	−0.0302 (2)	0.5490 (5)	0.0576 (15)	
H31A	0.9588	−0.0298	0.5714	0.069*	
H31B	1.0386	−0.0040	0.5841	0.069*	
H31C	1.0358	−0.0590	0.5751	0.069*	
C32	1.0325 (3)	−0.1150 (2)	0.3871 (7)	0.0645 (18)	
H32A	1.0527	−0.1142	0.4653	0.077*	
H32B	1.0743	−0.1183	0.3325	0.077*	
H32C	0.9977	−0.1411	0.3792	0.077*	
C33	0.8031 (5)	−0.0404 (3)	0.2966 (8)	0.114 (4)	
H33A	0.8048	−0.0450	0.3797	0.137*	
H33B	0.7804	−0.0675	0.2601	0.137*	
H33C	0.7725	−0.0131	0.2791	0.137*	
C34	0.8796 (4)	−0.0339 (2)	0.1222 (5)	0.0617 (16)	
H34A	0.8631	−0.0643	0.0960	0.074*	
H34B	0.9298	−0.0270	0.0904	0.074*	
H34C	0.8433	−0.0105	0.0965	0.074*	
C35	0.7242 (2)	0.09058 (17)	−0.0723 (4)	0.0329 (10)	
H35B	0.7426	0.1175	−0.1164	0.039*	
H35A	0.6832	0.1012	−0.0207	0.039*	
C36	0.6934 (2)	0.05468 (17)	−0.1537 (4)	0.0299 (9)	
C37	0.6355 (2)	0.06730 (17)	−0.2310 (4)	0.0302 (9)	
C38	0.6083 (2)	0.03406 (16)	−0.3057 (4)	0.0293 (9)	
H38A	0.5679	0.0422	−0.3554	0.035*	
C39	0.6374 (2)	−0.01113 (17)	−0.3120 (4)	0.0294 (9)	
C40	0.6040 (2)	−0.04378 (17)	−0.4024 (4)	0.0347 (10)	
C41	0.6475 (5)	−0.0890 (3)	−0.4092 (9)	0.134 (5)	
H41	0.6927	−0.0770	−0.4516	0.161*	
C42	0.6868 (5)	−0.1032 (2)	−0.3058 (7)	0.031 (2)	0.50
H42A	0.7196	−0.1295	−0.3273	0.037*	0.50
H42B	0.6478	−0.1155	−0.2529	0.037*	0.50
C42A	0.7184 (5)	−0.0948 (4)	−0.3478 (6)	0.041 (2)	0.50
C43	0.7335 (2)	−0.07171 (16)	−0.2398 (4)	0.0334 (10)	
C44	0.6960 (2)	−0.02344 (16)	−0.2365 (4)	0.0279 (9)	
C45	0.7219 (2)	0.00992 (17)	−0.1582 (4)	0.0300 (9)	
H45A	0.7609	0.0015	−0.1060	0.036*	
C46	0.6049 (2)	0.11608 (18)	−0.2334 (4)	0.0352 (10)	
H46A	0.6467	0.1377	−0.2464	0.042*	
H46B	0.5804	0.1231	−0.1600	0.042*	
H46C	0.5677	0.1190	−0.2955	0.042*	
C47	0.6092 (3)	−0.0211 (2)	−0.5203 (4)	0.0535 (14)	
H47A	0.6613	−0.0106	−0.5336	0.064*	
H47B	0.5747	0.0053	−0.5235	0.064*	
H47C	0.5951	−0.0435	−0.5795	0.064*	
C48	0.5201 (3)	−0.0526 (2)	−0.3749 (5)	0.0513 (14)	
H48A	0.4928	−0.0232	−0.3724	0.062*	
H48B	0.5159	−0.0680	−0.3004	0.062*	
H48C	0.4980	−0.0723	−0.4344	0.062*	
C49	0.6235 (3)	−0.1245 (2)	−0.4957 (6)	0.0561 (15)	
H49A	0.6555	−0.1220	−0.5641	0.067*	
H49B	0.5704	−0.1193	−0.5169	0.067*	
H49C	0.6289	−0.1553	−0.4625	0.067*	
C50	0.7243 (5)	−0.0956 (2)	−0.1244 (6)	0.079 (2)	
H50A	0.7436	−0.1271	−0.1295	0.094*	
H50B	0.6705	−0.0963	−0.1035	0.094*	
H50C	0.7527	−0.0786	−0.0658	0.094*	
C51	0.8185 (3)	−0.0658 (2)	−0.2623 (9)	0.081 (3)	
H51A	0.8259	−0.0508	−0.3368	0.097*	
H51B	0.8431	−0.0961	−0.2627	0.097*	
H51C	0.8408	−0.0467	−0.2019	0.097*	
C52	0.9070 (2)	0.06585 (18)	−0.2549 (4)	0.0338 (10)	
H52A	0.8759	0.0387	−0.2757	0.041*	
H52B	0.9567	0.0626	−0.2934	0.041*	
C53	0.8683 (2)	0.10894 (17)	−0.2996 (4)	0.0305 (9)	
C54	0.8123 (2)	0.10775 (17)	−0.3844 (4)	0.0313 (9)	
C55	0.7780 (2)	0.14903 (18)	−0.4168 (4)	0.0335 (10)	
H55A	0.7383	0.1478	−0.4717	0.040*	
C56	0.7988 (2)	0.19264 (17)	−0.3728 (4)	0.0304 (9)	
C57	0.7549 (3)	0.23592 (18)	−0.4114 (4)	0.0380 (10)	
C58	0.7837 (4)	0.2792 (2)	−0.3518 (9)	0.108 (4)	
H58	0.7598	0.2724	−0.2759	0.130*	
C59	0.8624 (4)	0.2790 (3)	−0.3103 (9)	0.039 (2)	0.50
H59A	0.8694	0.3068	−0.2625	0.047*	0.50
H59B	0.8958	0.2824	−0.3779	0.047*	0.50
C59A	0.8332 (5)	0.2762 (3)	−0.2507 (8)	0.049 (3)	0.50
C60	0.8902 (2)	0.23933 (15)	−0.2440 (4)	0.0368 (11)	
C61	0.8582 (2)	0.19386 (17)	−0.2922 (4)	0.0291 (9)	
C62	0.8905 (2)	0.15203 (17)	−0.2570 (4)	0.0309 (9)	
H62A	0.9297	0.1530	−0.2012	0.037*	
C63	0.7884 (3)	0.06276 (18)	−0.4417 (4)	0.0374 (11)	
H63A	0.7775	0.0396	−0.3828	0.045*	
H63B	0.8295	0.0517	−0.4911	0.045*	
H63C	0.7429	0.0680	−0.4880	0.045*	
C64	0.6701 (3)	0.2295 (2)	−0.3834 (5)	0.0552 (15)	
H64A	0.6638	0.2254	−0.3006	0.066*	
H64B	0.6507	0.2023	−0.4235	0.066*	
H64C	0.6418	0.2568	−0.4083	0.066*	
C65	0.7621 (4)	0.2418 (2)	−0.5409 (5)	0.0596 (16)	
H65A	0.7422	0.2143	−0.5794	0.072*	
H65B	0.8156	0.2459	−0.5612	0.072*	
H65C	0.7332	0.2689	−0.5653	0.072*	
C66	0.7428 (4)	0.3240 (2)	−0.3719 (6)	0.0616 (16)	
H66A	0.6880	0.3190	−0.3656	0.074*	
H66B	0.7549	0.3355	−0.4487	0.074*	
H66C	0.7589	0.3466	−0.3144	0.074*	
C67	0.9716 (4)	0.2446 (3)	−0.2856 (8)	0.093 (3)	
H67A	0.9927	0.2736	−0.2564	0.111*	
H67B	0.9724	0.2450	−0.3695	0.111*	
H67C	1.0021	0.2187	−0.2576	0.111*	
C68	0.8925 (4)	0.2384 (2)	−0.1146 (5)	0.0679 (18)	
H68C	0.9347	0.2189	−0.0892	0.082*	
H68B	0.8447	0.2258	−0.0852	0.082*	
H68A	0.8995	0.2697	−0.0854	0.082*	
O1	0.98051 (17)	0.13286 (13)	0.0048 (3)	0.0366 (7)	
H1	0.9671	0.1132	−0.0440	0.044*	
O2	0.84745 (17)	0.13860 (13)	0.1339 (3)	0.0332 (7)	
H2	0.8885	0.1431	0.0992	0.040*	
O3	0.78551 (16)	0.07181 (14)	−0.0052 (3)	0.0358 (7)	
H3	0.8017	0.0920	0.0401	0.043*	
O4	0.91858 (17)	0.06589 (13)	−0.1336 (3)	0.0337 (7)	
H4	0.8766	0.0685	−0.1002	0.040*	

### Atomic displacement parameters (Å^2^)

**Table:**

	*U*^11^	*U*^22^	*U*^33^	*U*^12^	*U*^13^	*U*^23^
C1	0.032 (2)	0.028 (2)	0.038 (2)	−0.0018 (18)	−0.0023 (18)	−0.0052 (19)
C2	0.0257 (19)	0.025 (2)	0.033 (2)	−0.0021 (16)	0.0001 (16)	−0.0006 (19)
C3	0.0247 (18)	0.026 (2)	0.036 (2)	−0.0008 (16)	0.0022 (17)	0.0011 (19)
C4	0.027 (2)	0.034 (2)	0.030 (2)	0.0040 (17)	0.0000 (17)	−0.0009 (18)
C5	0.029 (2)	0.031 (2)	0.034 (2)	−0.0047 (17)	0.0042 (18)	−0.0026 (19)
C6	0.032 (2)	0.032 (2)	0.044 (3)	−0.0023 (18)	−0.0012 (19)	−0.007 (2)
C7	0.076 (4)	0.065 (5)	0.120 (6)	0.036 (4)	−0.050 (4)	−0.063 (5)
C8	0.058 (4)	0.027 (3)	0.042 (4)	0.014 (3)	0.002 (3)	−0.008 (3)
C9	0.040 (2)	0.025 (2)	0.044 (3)	0.0023 (18)	−0.002 (2)	−0.0021 (19)
C10	0.0259 (19)	0.024 (2)	0.036 (2)	−0.0003 (16)	0.0027 (18)	−0.0003 (19)
C11	0.031 (2)	0.031 (2)	0.032 (2)	−0.0029 (18)	−0.0020 (18)	0.0028 (19)
C12	0.037 (2)	0.030 (2)	0.040 (3)	0.0010 (18)	0.001 (2)	−0.001 (2)
C13	0.073 (4)	0.073 (4)	0.039 (3)	−0.035 (3)	0.010 (3)	−0.013 (3)
C14	0.035 (2)	0.039 (3)	0.044 (3)	−0.0059 (19)	−0.002 (2)	−0.003 (2)
C15	0.049 (3)	0.045 (3)	0.066 (4)	0.003 (2)	−0.004 (3)	−0.026 (3)
C16	0.063 (3)	0.041 (3)	0.076 (4)	0.016 (3)	0.017 (3)	0.026 (3)
C17	0.053 (3)	0.037 (3)	0.149 (8)	0.016 (3)	0.043 (5)	0.019 (4)
C18	0.033 (2)	0.030 (2)	0.038 (3)	0.0015 (17)	−0.006 (2)	−0.004 (2)
C19	0.030 (2)	0.025 (2)	0.030 (2)	−0.0013 (17)	0.0046 (16)	−0.0031 (18)
C20	0.029 (2)	0.029 (2)	0.034 (2)	−0.0026 (17)	0.0026 (17)	−0.0029 (19)
C21	0.030 (2)	0.033 (2)	0.034 (2)	−0.0037 (18)	−0.0030 (17)	0.0020 (19)
C22	0.030 (2)	0.028 (2)	0.035 (2)	−0.0001 (17)	0.0066 (17)	−0.0001 (19)
C23	0.033 (2)	0.029 (2)	0.048 (3)	0.0032 (18)	0.0016 (19)	0.002 (2)
C24	0.069 (4)	0.038 (4)	0.187 (9)	0.018 (3)	−0.062 (5)	−0.040 (5)
C25	0.062 (6)	0.026 (5)	0.039 (6)	−0.016 (4)	0.028 (5)	0.000 (4)
C25A	0.077 (8)	0.017 (5)	0.059 (7)	−0.011 (5)	−0.040 (6)	0.017 (5)
C26	0.043 (2)	0.034 (3)	0.049 (3)	−0.005 (2)	−0.003 (2)	−0.002 (2)
C27	0.033 (2)	0.026 (2)	0.034 (2)	−0.0042 (17)	0.0059 (17)	0.0027 (19)
C28	0.0267 (19)	0.036 (3)	0.034 (2)	−0.0023 (17)	0.001 (2)	−0.002 (2)
C29	0.034 (2)	0.030 (2)	0.034 (2)	0.0001 (18)	−0.0023 (18)	−0.0035 (19)
C30	0.036 (2)	0.044 (3)	0.060 (3)	0.008 (2)	0.000 (2)	0.013 (2)
C31	0.059 (3)	0.055 (3)	0.059 (4)	0.018 (3)	0.013 (3)	0.023 (3)
C32	0.052 (3)	0.032 (3)	0.109 (6)	0.009 (2)	−0.010 (3)	−0.007 (3)
C33	0.114 (6)	0.108 (7)	0.121 (7)	−0.087 (6)	0.071 (6)	−0.069 (6)
C34	0.087 (4)	0.048 (3)	0.050 (3)	−0.014 (3)	0.004 (3)	−0.014 (3)
C35	0.032 (2)	0.034 (2)	0.033 (2)	0.0017 (18)	−0.0040 (18)	−0.0043 (19)
C36	0.027 (2)	0.027 (2)	0.036 (2)	−0.0023 (16)	0.0021 (17)	−0.0024 (19)
C37	0.0272 (19)	0.028 (2)	0.035 (2)	0.0018 (16)	0.0021 (17)	0.0015 (19)
C38	0.025 (2)	0.027 (2)	0.036 (2)	−0.0012 (16)	−0.0034 (17)	0.0002 (18)
C39	0.027 (2)	0.027 (2)	0.035 (2)	−0.0023 (17)	0.0018 (17)	−0.0032 (19)
C40	0.034 (2)	0.029 (2)	0.041 (3)	0.0016 (18)	−0.0036 (19)	−0.007 (2)
C41	0.149 (8)	0.076 (5)	0.178 (9)	0.075 (5)	−0.139 (8)	−0.087 (6)
C42	0.028 (4)	0.016 (4)	0.048 (6)	−0.005 (3)	0.005 (4)	0.000 (4)
C42A	0.054 (7)	0.027 (5)	0.042 (6)	0.005 (5)	−0.004 (5)	−0.004 (5)
C43	0.033 (2)	0.026 (2)	0.042 (3)	0.0058 (18)	0.005 (2)	0.0029 (19)
C44	0.0260 (18)	0.024 (2)	0.034 (2)	−0.0011 (15)	0.0018 (18)	0.0029 (18)
C45	0.0259 (18)	0.027 (2)	0.037 (2)	0.0000 (17)	−0.0016 (17)	0.0017 (19)
C46	0.035 (2)	0.031 (2)	0.039 (3)	0.0028 (18)	−0.0028 (19)	−0.002 (2)
C47	0.061 (3)	0.064 (4)	0.036 (3)	−0.029 (3)	0.002 (2)	−0.010 (3)
C48	0.045 (3)	0.060 (4)	0.049 (3)	−0.024 (2)	−0.003 (2)	−0.001 (3)
C49	0.060 (3)	0.035 (3)	0.073 (4)	0.009 (2)	−0.022 (3)	−0.017 (3)
C50	0.121 (6)	0.048 (4)	0.067 (4)	0.049 (4)	0.027 (4)	0.021 (3)
C51	0.037 (3)	0.031 (3)	0.174 (8)	0.011 (2)	0.024 (4)	0.005 (4)
C52	0.037 (2)	0.033 (2)	0.032 (2)	0.0007 (18)	0.000 (2)	0.0001 (19)
C53	0.0266 (19)	0.031 (2)	0.034 (2)	0.0008 (18)	0.0008 (17)	−0.0028 (19)
C54	0.030 (2)	0.031 (2)	0.033 (2)	−0.0043 (17)	0.0017 (17)	−0.0010 (19)
C55	0.030 (2)	0.036 (2)	0.035 (2)	−0.0023 (18)	−0.0030 (17)	0.000 (2)
C56	0.029 (2)	0.032 (2)	0.030 (2)	0.0026 (17)	0.0023 (17)	0.0041 (19)
C57	0.037 (2)	0.036 (2)	0.042 (3)	0.006 (2)	−0.0024 (19)	0.002 (2)
C58	0.111 (6)	0.034 (3)	0.179 (9)	0.028 (4)	−0.099 (6)	−0.031 (4)
C59	0.050 (6)	0.030 (5)	0.039 (6)	−0.003 (4)	0.014 (5)	−0.004 (4)
C59A	0.046 (6)	0.039 (6)	0.060 (8)	0.001 (5)	−0.005 (6)	−0.012 (5)
C60	0.041 (2)	0.025 (3)	0.045 (3)	−0.0048 (18)	−0.007 (2)	−0.002 (2)
C61	0.029 (2)	0.028 (2)	0.030 (2)	−0.0010 (17)	0.0030 (17)	−0.0017 (18)
C62	0.0284 (19)	0.030 (2)	0.034 (2)	−0.0001 (16)	−0.0054 (19)	−0.0024 (19)
C63	0.041 (2)	0.032 (3)	0.039 (3)	−0.0077 (19)	−0.008 (2)	−0.001 (2)
C64	0.043 (3)	0.056 (4)	0.067 (4)	0.016 (2)	0.009 (3)	0.018 (3)
C65	0.066 (3)	0.066 (4)	0.047 (3)	0.026 (3)	0.010 (3)	0.018 (3)
C66	0.065 (4)	0.031 (3)	0.089 (5)	0.006 (3)	−0.017 (3)	0.001 (3)
C67	0.078 (5)	0.090 (6)	0.110 (6)	−0.058 (4)	0.039 (4)	−0.048 (5)
C68	0.101 (5)	0.052 (4)	0.051 (3)	−0.020 (3)	0.005 (3)	−0.023 (3)
O1	0.0364 (17)	0.0336 (17)	0.0399 (19)	0.0002 (13)	−0.0081 (14)	−0.0059 (14)
O2	0.0315 (14)	0.0324 (17)	0.0359 (16)	0.0019 (13)	−0.0026 (12)	−0.0010 (14)
O3	0.0330 (15)	0.0353 (17)	0.0390 (18)	0.0016 (12)	−0.0091 (13)	−0.0065 (14)
O4	0.0319 (14)	0.0317 (17)	0.0377 (17)	0.0025 (13)	−0.0053 (13)	0.0001 (14)

### Geometric parameters (Å, °)

**Table:**

C1—O1	1.428 (5)	C35—H35B	0.9900
C1—C2	1.503 (6)	C35—H35A	0.9900
C1—H1B	0.9900	C36—C45	1.394 (6)
C1—H1A	0.9900	C36—C37	1.411 (6)
C2—C11	1.397 (6)	C37—C38	1.386 (6)
C2—C3	1.405 (6)	C37—C46	1.515 (6)
C3—C4	1.376 (6)	C38—C39	1.410 (6)
C3—C12	1.519 (6)	C38—H38A	0.9500
C4—C5	1.405 (6)	C39—C44	1.404 (6)
C4—H4A	0.9500	C39—C40	1.536 (6)
C5—C10	1.405 (6)	C40—C41	1.521 (7)
C5—C6	1.548 (6)	C40—C47	1.528 (7)
C6—C13	1.515 (8)	C40—C48	1.535 (7)
C6—C14	1.535 (6)	C41—C42	1.451 (5)
C6—C7	1.548 (8)	C41—C49	1.504 (8)
C7—C8	1.491 (5)	C41—H41	1.0000
C7—C15	1.514 (7)	C42—C43	1.452 (5)
C7—H7	1.0000	C42—H42A	0.9900
C8—C9	1.492 (5)	C42—H42B	0.9900
C8—H8A	0.9900	C42A—C43	1.452 (5)
C8—H8B	0.9900	C43—C50	1.523 (8)
C8A—C9	1.492 (5)	C43—C51	1.530 (7)
C9—C17	1.530 (7)	C43—C44	1.550 (6)
C9—C16	1.532 (7)	C44—C45	1.407 (6)
C9—C10	1.535 (6)	C45—H45A	0.9500
C10—C11	1.406 (6)	C46—H46A	0.9800
C11—H11A	0.9500	C46—H46B	0.9800
C12—H12A	0.9800	C46—H46C	0.9800
C12—H12B	0.9800	C47—H47A	0.9800
C12—H12C	0.9800	C47—H47B	0.9800
C13—H13A	0.9800	C47—H47C	0.9800
C13—H13B	0.9800	C48—H48A	0.9800
C13—H13C	0.9800	C48—H48B	0.9800
C14—H14A	0.9800	C48—H48C	0.9800
C14—H14B	0.9800	C49—H49A	0.9800
C14—H14C	0.9800	C49—H49B	0.9800
C15—H15A	0.9800	C49—H49C	0.9800
C15—H15B	0.9800	C50—H50A	0.9800
C15—H15C	0.9800	C50—H50B	0.9800
C16—H16A	0.9800	C50—H50C	0.9800
C16—H16B	0.9800	C51—H51A	0.9800
C16—H16C	0.9800	C51—H51B	0.9800
C17—H17A	0.9800	C51—H51C	0.9800
C17—H17B	0.9800	C52—O4	1.431 (5)
C17—H17C	0.9800	C52—C53	1.517 (6)
C18—O2	1.432 (5)	C52—H52A	0.9900
C18—C19	1.525 (6)	C52—H52B	0.9900
C18—H18B	0.9900	C53—C54	1.400 (6)
C18—H18A	0.9900	C53—C62	1.401 (6)
C19—C28	1.395 (6)	C54—C55	1.394 (7)
C19—C20	1.412 (6)	C54—C63	1.526 (6)
C20—C21	1.385 (6)	C55—C56	1.414 (7)
C20—C29	1.515 (6)	C55—H55A	0.9500
C21—C22	1.413 (6)	C56—C61	1.408 (6)
C21—H21A	0.9500	C56—C57	1.543 (6)
C22—C27	1.407 (6)	C57—C58	1.521 (8)
C22—C23	1.550 (6)	C57—C65	1.525 (7)
C23—C24	1.510 (8)	C57—C64	1.542 (7)
C23—C30	1.526 (6)	C58—C59	1.470 (5)
C23—C31	1.527 (8)	C58—C66	1.505 (8)
C24—C25	1.470 (5)	C58—H58	1.0000
C24—C32	1.517 (8)	C59—C60	1.471 (5)
C24—H24	1.0000	C59—H59A	0.9900
C25—C26	1.471 (5)	C59—H59B	0.9900
C25—H25A	0.9900	C59A—C60	1.471 (5)
C25—H25B	0.9900	C60—C68	1.510 (8)
C25A—C26	1.471 (5)	C60—C67	1.524 (8)
C26—C34	1.503 (8)	C60—C61	1.541 (6)
C26—C33	1.532 (8)	C61—C62	1.403 (6)
C26—C27	1.543 (7)	C62—H62A	0.9500
C27—C28	1.393 (7)	C63—H63A	0.9800
C28—H28A	0.9500	C63—H63B	0.9800
C29—H29A	0.9800	C63—H63C	0.9800
C29—H29B	0.9800	C64—H64A	0.9800
C29—H29C	0.9800	C64—H64B	0.9800
C30—H30A	0.9800	C64—H64C	0.9800
C30—H30B	0.9800	C65—H65A	0.9800
C30—H30C	0.9800	C65—H65B	0.9800
C31—H31A	0.9800	C65—H65C	0.9800
C31—H31B	0.9800	C66—H66A	0.9800
C31—H31C	0.9800	C66—H66B	0.9800
C32—H32A	0.9800	C66—H66C	0.9800
C32—H32B	0.9800	C67—H67A	0.9800
C32—H32C	0.9800	C67—H67B	0.9800
C33—H33A	0.9800	C67—H67C	0.9800
C33—H33B	0.9800	C68—H68C	0.9800
C33—H33C	0.9800	C68—H68B	0.9800
C34—H34A	0.9800	C68—H68A	0.9800
C34—H34B	0.9800	O1—H1	0.8400
C34—H34C	0.9800	O2—H2	0.8400
C35—O3	1.442 (5)	O3—H3	0.8400
C35—C36	1.511 (6)	O4—H4	0.8400
			
O1—C1—C2	111.8 (4)	C36—C35—H35B	109.5
O1—C1—H1B	109.3	O3—C35—H35A	109.5
C2—C1—H1B	109.3	C36—C35—H35A	109.5
O1—C1—H1A	109.3	H35B—C35—H35A	108.1
C2—C1—H1A	109.3	C45—C36—C37	118.6 (4)
H1B—C1—H1A	107.9	C45—C36—C35	122.4 (4)
C11—C2—C3	118.5 (4)	C37—C36—C35	118.9 (4)
C11—C2—C1	122.1 (4)	C38—C37—C36	118.2 (4)
C3—C2—C1	119.4 (4)	C38—C37—C46	121.0 (4)
C4—C3—C2	118.7 (4)	C36—C37—C46	120.8 (4)
C4—C3—C12	120.7 (4)	C37—C38—C39	123.7 (4)
C2—C3—C12	120.6 (4)	C37—C38—H38A	118.2
C3—C4—C5	123.7 (4)	C39—C38—H38A	118.2
C3—C4—H4A	118.2	C44—C39—C38	118.1 (4)
C5—C4—H4A	118.2	C44—C39—C40	123.8 (4)
C4—C5—C10	118.0 (4)	C38—C39—C40	118.0 (4)
C4—C5—C6	117.6 (4)	C41—C40—C47	107.1 (6)
C10—C5—C6	124.4 (4)	C41—C40—C48	110.7 (6)
C13—C6—C14	109.9 (4)	C47—C40—C48	108.6 (4)
C13—C6—C5	108.5 (4)	C41—C40—C39	111.9 (4)
C14—C6—C5	109.1 (4)	C47—C40—C39	109.2 (4)
C13—C6—C7	111.8 (5)	C48—C40—C39	109.3 (4)
C14—C6—C7	106.4 (5)	C42—C41—C49	119.8 (6)
C5—C6—C7	111.1 (4)	C42—C41—C40	116.3 (6)
C8—C7—C15	116.0 (5)	C49—C41—C40	118.9 (4)
C8—C7—C6	116.1 (5)	C42—C41—H41	97.4
C15—C7—C6	115.5 (5)	C49—C41—H41	97.4
C8—C7—H7	101.9	C40—C41—H41	97.4
C15—C7—H7	101.9	C41—C42—C43	122.2 (5)
C6—C7—H7	101.9	C41—C42—H42A	106.8
C7—C8—C9	116.8 (5)	C43—C42—H42A	106.8
C7—C8—H8A	108.1	C41—C42—H42B	106.8
C9—C8—H8A	108.1	C43—C42—H42B	106.8
C7—C8—H8B	108.1	H42A—C42—H42B	106.6
C9—C8—H8B	108.1	C42A—C43—C50	122.5 (7)
H8A—C8—H8B	107.3	C42—C43—C50	97.0 (6)
C8A—C9—C17	129.8 (10)	C42A—C43—C51	94.7 (6)
C8—C9—C17	101.0 (5)	C42—C43—C51	122.3 (6)
C8A—C9—C16	81.3 (6)	C50—C43—C51	107.9 (6)
C8—C9—C16	117.2 (5)	C42A—C43—C44	111.1 (5)
C17—C9—C16	106.4 (5)	C42—C43—C44	109.9 (4)
C8A—C9—C10	112.2 (7)	C50—C43—C44	110.1 (4)
C8—C9—C10	110.4 (4)	C51—C43—C44	108.7 (4)
C17—C9—C10	110.8 (4)	C39—C44—C45	118.1 (4)
C16—C9—C10	110.4 (4)	C39—C44—C43	121.9 (4)
C5—C10—C11	118.3 (4)	C45—C44—C43	119.9 (4)
C5—C10—C9	121.8 (4)	C36—C45—C44	123.3 (4)
C11—C10—C9	119.9 (4)	C36—C45—H45A	118.4
C2—C11—C10	122.8 (4)	C44—C45—H45A	118.4
C2—C11—H11A	118.6	C37—C46—H46A	109.5
C10—C11—H11A	118.6	C37—C46—H46B	109.5
C3—C12—H12A	109.5	H46A—C46—H46B	109.5
C3—C12—H12B	109.5	C37—C46—H46C	109.5
H12A—C12—H12B	109.5	H46A—C46—H46C	109.5
C3—C12—H12C	109.5	H46B—C46—H46C	109.5
H12A—C12—H12C	109.5	C40—C47—H47A	109.5
H12B—C12—H12C	109.5	C40—C47—H47B	109.5
C6—C13—H13A	109.5	H47A—C47—H47B	109.5
C6—C13—H13B	109.5	C40—C47—H47C	109.5
H13A—C13—H13B	109.5	H47A—C47—H47C	109.5
C6—C13—H13C	109.5	H47B—C47—H47C	109.5
H13A—C13—H13C	109.5	C40—C48—H48A	109.5
H13B—C13—H13C	109.5	C40—C48—H48B	109.5
C6—C14—H14A	109.5	H48A—C48—H48B	109.5
C6—C14—H14B	109.5	C40—C48—H48C	109.5
H14A—C14—H14B	109.5	H48A—C48—H48C	109.5
C6—C14—H14C	109.5	H48B—C48—H48C	109.5
H14A—C14—H14C	109.5	C41—C49—H49A	109.5
H14B—C14—H14C	109.5	C41—C49—H49B	109.5
C7—C15—H15A	109.5	H49A—C49—H49B	109.5
C7—C15—H15B	109.5	C41—C49—H49C	109.5
H15A—C15—H15B	109.5	H49A—C49—H49C	109.5
C7—C15—H15C	109.5	H49B—C49—H49C	109.5
H15A—C15—H15C	109.5	C43—C50—H50A	109.5
H15B—C15—H15C	109.5	C43—C50—H50B	109.5
C9—C16—H16A	109.5	H50A—C50—H50B	109.5
C9—C16—H16B	109.5	C43—C50—H50C	109.5
H16A—C16—H16B	109.5	H50A—C50—H50C	109.5
C9—C16—H16C	109.5	H50B—C50—H50C	109.5
H16A—C16—H16C	109.5	C43—C51—H51A	109.5
H16B—C16—H16C	109.5	C43—C51—H51B	109.5
C9—C17—H17A	109.5	H51A—C51—H51B	109.5
C9—C17—H17B	109.5	C43—C51—H51C	109.5
H17A—C17—H17B	109.5	H51A—C51—H51C	109.5
C9—C17—H17C	109.5	H51B—C51—H51C	109.5
H17A—C17—H17C	109.5	O4—C52—C53	113.8 (4)
H17B—C17—H17C	109.5	O4—C52—H52A	108.8
O2—C18—C19	113.2 (4)	C53—C52—H52A	108.8
O2—C18—H18B	108.9	O4—C52—H52B	108.8
C19—C18—H18B	108.9	C53—C52—H52B	108.8
O2—C18—H18A	108.9	H52A—C52—H52B	107.7
C19—C18—H18A	108.9	C54—C53—C62	118.1 (4)
H18B—C18—H18A	107.8	C54—C53—C52	122.7 (4)
C28—C19—C20	118.5 (4)	C62—C53—C52	119.2 (4)
C28—C19—C18	119.8 (4)	C55—C54—C53	118.5 (4)
C20—C19—C18	121.7 (4)	C55—C54—C63	119.8 (4)
C21—C20—C19	117.4 (4)	C53—C54—C63	121.7 (4)
C21—C20—C29	120.7 (4)	C54—C55—C56	123.9 (4)
C19—C20—C29	122.0 (4)	C54—C55—H55A	118.0
C20—C21—C22	124.8 (4)	C56—C55—H55A	118.0
C20—C21—H21A	117.6	C61—C56—C55	117.3 (4)
C22—C21—H21A	117.6	C61—C56—C57	123.2 (4)
C27—C22—C21	116.9 (4)	C55—C56—C57	119.5 (4)
C27—C22—C23	124.0 (4)	C58—C57—C65	109.5 (6)
C21—C22—C23	119.1 (4)	C58—C57—C56	111.7 (4)
C24—C23—C30	109.4 (5)	C65—C57—C56	109.7 (4)
C24—C23—C31	110.6 (5)	C58—C57—C64	109.0 (5)
C30—C23—C31	108.1 (4)	C65—C57—C64	107.7 (4)
C24—C23—C22	110.8 (4)	C56—C57—C64	109.1 (4)
C30—C23—C22	108.7 (4)	C59—C58—C66	120.4 (6)
C31—C23—C22	109.1 (4)	C59—C58—C57	117.5 (6)
C25—C24—C23	120.2 (6)	C66—C58—C57	118.8 (5)
C25—C24—C32	117.7 (6)	C59—C58—H58	96.0
C23—C24—C32	118.0 (5)	C66—C58—H58	96.0
C25—C24—H24	96.8	C57—C58—H58	96.0
C23—C24—H24	96.8	C58—C59—C60	119.4 (5)
C32—C24—H24	96.8	C58—C59—H59A	107.5
C24—C25—C26	118.4 (5)	C60—C59—H59A	107.5
C24—C25—H25A	107.7	C58—C59—H59B	107.5
C26—C25—H25A	107.7	C60—C59—H59B	107.5
C24—C25—H25B	107.7	H59A—C59—H59B	107.0
C26—C25—H25B	107.7	C59—C60—C68	123.4 (6)
H25A—C25—H25B	107.1	C59A—C60—C68	94.9 (6)
C25A—C26—C34	123.5 (7)	C59—C60—C67	93.9 (6)
C25—C26—C34	94.8 (5)	C59A—C60—C67	123.6 (7)
C25A—C26—C33	91.4 (7)	C68—C60—C67	107.2 (5)
C25—C26—C33	124.4 (7)	C59—C60—C61	110.8 (5)
C34—C26—C33	107.5 (5)	C59A—C60—C61	110.7 (5)
C25A—C26—C27	111.8 (5)	C68—C60—C61	111.0 (4)
C25—C26—C27	109.6 (5)	C67—C60—C61	108.4 (4)
C34—C26—C27	111.5 (4)	C62—C61—C56	118.4 (4)
C33—C26—C27	108.1 (4)	C62—C61—C60	119.0 (4)
C28—C27—C22	118.6 (4)	C56—C61—C60	122.5 (4)
C28—C27—C26	119.8 (4)	C53—C62—C61	123.7 (4)
C22—C27—C26	121.6 (4)	C53—C62—H62A	118.2
C27—C28—C19	123.7 (4)	C61—C62—H62A	118.2
C27—C28—H28A	118.1	C54—C63—H63A	109.5
C19—C28—H28A	118.1	C54—C63—H63B	109.5
C20—C29—H29A	109.5	H63A—C63—H63B	109.5
C20—C29—H29B	109.5	C54—C63—H63C	109.5
H29A—C29—H29B	109.5	H63A—C63—H63C	109.5
C20—C29—H29C	109.5	H63B—C63—H63C	109.5
H29A—C29—H29C	109.5	C57—C64—H64A	109.5
H29B—C29—H29C	109.5	C57—C64—H64B	109.5
C23—C30—H30A	109.5	H64A—C64—H64B	109.5
C23—C30—H30B	109.5	C57—C64—H64C	109.5
H30A—C30—H30B	109.5	H64A—C64—H64C	109.5
C23—C30—H30C	109.5	H64B—C64—H64C	109.5
H30A—C30—H30C	109.5	C57—C65—H65A	109.5
H30B—C30—H30C	109.5	C57—C65—H65B	109.5
C23—C31—H31A	109.5	H65A—C65—H65B	109.5
C23—C31—H31B	109.5	C57—C65—H65C	109.5
H31A—C31—H31B	109.5	H65A—C65—H65C	109.5
C23—C31—H31C	109.5	H65B—C65—H65C	109.5
H31A—C31—H31C	109.5	C58—C66—H66A	109.5
H31B—C31—H31C	109.5	C58—C66—H66B	109.5
C24—C32—H32A	109.5	H66A—C66—H66B	109.5
C24—C32—H32B	109.5	C58—C66—H66C	109.5
H32A—C32—H32B	109.5	H66A—C66—H66C	109.5
C24—C32—H32C	109.5	H66B—C66—H66C	109.5
H32A—C32—H32C	109.5	C60—C67—H67A	109.5
H32B—C32—H32C	109.5	C60—C67—H67B	109.5
C26—C33—H33A	109.5	H67A—C67—H67B	109.5
C26—C33—H33B	109.5	C60—C67—H67C	109.5
H33A—C33—H33B	109.5	H67A—C67—H67C	109.5
C26—C33—H33C	109.5	H67B—C67—H67C	109.5
H33A—C33—H33C	109.5	C60—C68—H68C	109.5
H33B—C33—H33C	109.5	C60—C68—H68B	109.5
C26—C34—H34A	109.5	H68C—C68—H68B	109.5
C26—C34—H34B	109.5	C60—C68—H68A	109.5
H34A—C34—H34B	109.5	H68C—C68—H68A	109.5
C26—C34—H34C	109.5	H68B—C68—H68A	109.5
H34A—C34—H34C	109.5	C1—O1—H1	109.5
H34B—C34—H34C	109.5	C18—O2—H2	109.5
O3—C35—C36	110.5 (4)	C35—O3—H3	109.5
O3—C35—H35B	109.5	C52—O4—H4	109.5

### Hydrogen-bond geometry (Å, °)

**Table:**

Cg4, Cg10, Cg14 and Cg17 are the centroids of the C19–C22/C27/C28, C36–C39/C44/C45, C53–C56/C61/C62 and C2–C5/C10/C11 rings, respectively.

**Table:**

*D*—H···*A*	*D*—H	H···*A*	*D*···*A*	*D*—H···*A*
O1—H1···O4	0.84	1.93	2.753 (5)	168
O2—H2···O1	0.84	1.98	2.794 (4)	162
O3—H3···O2	0.84	1.92	2.7545)	174
O4—H4···O3	0.84	1.95	2.790 (4)	173
C29—H29A···Cg14^i^	0.98	2.83	3.632 (4)	140
C29—H29B···Cg17	0.98	2.58	3.466 (5)	151
C63—H63A···Cg10	0.98	2.69	3.471 (5)	137
C63—H63B···Cg4^ii^	0.98	2.72	3.641 (5)	157

Symmetry codes:  (i) *x*, *y*, *z*+1; (ii) *x*, *y*, *z*−1.
